# Editorial: Host-Pathogen Interactions During Pneumococcal Infection

**DOI:** 10.3389/fcimb.2021.752959

**Published:** 2021-10-25

**Authors:** Masaya Yamaguchi, Yuki Kinjo, Victor Nizet

**Affiliations:** ^1^ Department of Oral and Molecular Microbiology, Osaka University Graduate School of Dentistry, Suita, Japan; ^2^ Department of Bacteriology, The Jikei University School of Medicine, Tokyo, Japan; ^3^ Jikei Center for Biofilm Science and Technology, The Jikei University School of Medicine, Tokyo, Japan; ^4^ Department of Intelligent Network for Infection Control, Tohoku University Graduate School of Medicine, Sendai, Japan; ^5^ Department of Pediatrics, University of California (UC), San Diego, La Jolla, CA, United States; ^6^ Skaggs School of Pharmacy and Pharmaceutical Sciences, University of California (UC), San Diego, La Jolla, CA, United States; ^7^ Biomedical Sciences Graduate Program, University of California (UC), San Diego, La Jolla, CA, United States

**Keywords:** *Streptococcus pneumoniae*, host immunity, virulence factor, inflammation, recognition mechanism

*Streptococcus pneumoniae*, a Gram-positive bacterium belonging to the mitis group, colonizes the human nasopharynx and is a leading cause of community-acquired pneumonia, meningitis, and bacteremia worldwide. Although approximately 20-40% of children carry this organism in their nasopharynx without clinical symptoms, *S. pneumoniae* is estimated to be responsible for the deaths of approximately 1,190,000 people annually from lower respiratory infections (Bogaert et al., 2004; Otsuka et al., 2013; GBD 2016 Lower Respiratory Infections Collaborators, 2018). In addition, antibiotic selective pressure has caused resistant pneumococcal clones to emerge and expand throughout the world, prompting the World Health Organization to list *S. pneumoniae* as one of 12 priority antibiotic-resistant pathogens (WHO, 2017). Data from active bacterial core surveillance obtained from 2009 to 2013 and presented by the U.S. Centers for Disease Control indicate that widespread availability of pneumococcal conjugate vaccines is effective in countering emerging antibiotic resistance (CDC, 2019). However, while such vaccines target major pneumococcal capsular serotypes, only a subset of the 100 different known serotypes are covered, applying a selective pressure for niche replacement such that the prevalence of non-vaccine serotypes of *S. pneumoniae* has been increasing worldwide (Golubchik et al., 2012). To establish novel effective control strategies, it is important to elucidate the detailed pathogenic process of pneumococcal infection.

For this *Frontiers in Cellular and Infection Microbiology* Research Topic, we collected 13 papers, including six original research studies, six review (full and mini) papers, and one perspective. Two review papers and two original studies describing fundamental microbiological characteristic of *S. pneumoniae* related to infection serve as an excellent starting point. Sanchez-Rosario and Johnson described differences between historical and modern pneumococcal growth media, highlighting key components that are necessary for cultivation/growth enhancement and their effects on bacterial phenotypes and experimental outcomes. Such precise knowledge of media used for bacterial growth is essential for pneumococcal investigators to improve the quality and comparability of ongoing research. Luck et al. reviewed the role of the pneumococcal polysaccharide capsule related to pathogenesis and the particular importance of serotype 3, with focus on capsule synthesis, localization, and biochemical and physiological properties, as well as mechanisms of vaccine escape. On the topic of serotype replacement by pneumococcal polysaccharide vaccines, immunogenic protein antigens are being explored as an alternative to multivalent polysaccharide vaccines, with the pneumococcal surface protein A (PspA) protein a particularly promising vaccine antigen candidate (Briles et al., 2019). PspA proteins are classified into three families that are further discriminated into six clades (Hollingshead et al., 2000). In analysis of 1,939 strains isolated from cases of adult invasive pneumococcal disease in Japan, Chang et al. determined the PspA clades of 1,928 and identified new PspA clades for another four. Those findings can help in guiding the design of effective multi-valent PspA vaccines in the near future. Lastly, Ali et al. examined the role of the pneumococcal serine proteases HtrA, PrtA, SFP, and CbpG in human epithelial cell adherence and mouse nasopharyngeal colonization. Their results showed that protease deficiency reduced pneumococcal colonization, suggesting this family of virulence determinants may be useful therapeutic targets to prevent colonization and transmission.

Next, one perspective, one mini-review, and one original article that centered on novel aspects of pneumococcal host-pathogen interactions are included. Tuomanen reported that three leading bacteria agents of childhood meningitis, *S. pneumoniae*, *Haemophilus influenzae*, and *Neisseria meningitidis*, share a similar strategy for invasion of the blood-brain barrier endothelium including utilization of the host platelet-activating factor (PAF) and laminin receptors. This commonality in pathogenic mechanism may provide a template for development of a broadly cross-protective meningitis vaccine. As part of the infectious process, host neutrophils play important roles as part of the first line of defense against pathogens (Kinjo et al., 2011; Dohrmann et al., 2016). However, excessive neutrophil activation causes destruction of host tissues. Domon and Terao summarized the way how neutrophilic inflammation can have detrimental effects in pneumococcal pneumonia, with a particular focus on neutrophil elastase, which cleaves a variety of host proteins to cause lung injury and barrier compromise. *S. pneumoniae* can degrade, transport, and metabolize various host glycans (Hobbs et al., 2018). The pneumococcal sialidase NanA is a multi-functional virulence factor shown to contribute to otitis media, meningitis, and exaggerated inflammatory responses in small animal infection models (Tong et al., 2000; Uchiyama et al., 2009; Chang et al., 2012). Evolutionary analyses reveal the encoding pneumococcal *nanA* gene is under considerable negative selection pressure (Yamaguchi et al., 2016; Yamaguchi et al., 2019; Yamaguchi et al., 2020). In our Research Topic, Tseng et al. reported a novel virulence role of NanA in pneumococcal pathogenesis, in which the desialylation it causes impairs the interaction of sialic acid-binding Ig-like lectin 5 (Siglec-5) and Toll-like receptor 2 (TLR-2), provoking excessive inflammation and cytotoxicity in infected macrophages.

Two reviews, a mini-review, and a research article explored clinicopathological correlates of disease outcome in pneumococcal infection. Increased mortality risk in pneumococcal infection can be seen in patients that experience an imbalance of cytokine responses and heightened inflammatory status (Franceschi and Campisi, 2014; Ferrucci and Fabbri, 2018). The details of this state were described by Weight et al. along with analysis of corresponding mucosal epithelial and innate immune alterations, highlighting how immunologic changes of aging strongly increase risk of adverse disease outcome. Co-infection with influenza virus is another major factor that increases the potential for severe or lethal pneumococcal infection. Sender et al. described how a preceding influenza infection modulates host immune response and how *S. pneumoniae* senses and adapts to the modified environment. Influenza virus co-infection also enhances pneumococcal transmission. Morimura et al. summarized that relationship and introduce the remarkable utility of new animal models to characterize this key factor of pneumococcal infection that poses a serious threat to public health. Finally, Murakami et al. demonstrated that cigarette smoke exposure significantly promotes pneumococcal transmission by enhancing bacterial shedding from the host, increasing the likelihood of pneumococcal colonization in contacts. This study was the first to experimentally demonstrate the importance of an environmental factor (cigarette smoke) for pneumococcal transmission.

Finally, two original papers that reported novel therapeutic candidates were reviewed. In a “drugs from bugs” approach, Li et al. showed that stimulation of macrophages with *S. pneumoniae* endopeptidase O (PepO), a multifunctional pneumococcal virulence protein, enhanced phagocytic clearance of *Staphylococcus aureus* and *S. pneumoniae.* The work by Nakakubo et al. showed that Hochu-Ekki-to, a traditional Japanese herbal medicine cocktail, accelerates clearance of pneumococcal colonization through macrophage activation and increased IL-17A production. To best address infectious challenges in the era of ever increasing antibiotic resistance, it will be necessary to continue to explore such innovative immune boosting therapeutic strategies.

In sum, the 13 papers of our Research Topic provide an exciting addition to the literature on host-pathogen interactions during pneumococcal infection, including a number of important new scientific findings and thoughtful synthesis of emerging paradigms. We hope these contributions will help investigators in the field continue to propel forward the science and clinical therapeutics of this foremost of human bacterial pathogens.

## Author Contributions

All authors listed have made substantial, direct, and intellectual contributions to the work, and approved the final version for publication.

## Conflict of Interest

The authors declare that the research was conducted in the absence of any commercial or financial relationships that could be construed as a potential conflict of interest.

## Publisher’s Note

All claims expressed in this article are solely those of the authors and do not necessarily represent those of their affiliated organizations, or those of the publisher, the editors and the reviewers. Any product that may be evaluated in this article, or claim that may be made by its manufacturer, is not guaranteed or endorsed by the publisher.
